# Experiential and Strategic Emotional Intelligence Are Implicated When Inhibiting Affective and Non-Affective Distractors: Findings from Three Emotional Flanker N-Back Tasks

**DOI:** 10.3390/jintelligence9010012

**Published:** 2021-03-01

**Authors:** Ming D. Lim, Damian P. Birney

**Affiliations:** School of Psychology, University of Sydney, Sydney, NSW 2006, Australia; damian.birney@sydney.edu.au

**Keywords:** emotional intelligence, MSCEIT, *n*-back task, updating, inhibition, affective distractors, accuracy rates, response latencies

## Abstract

Emotional intelligence (EI) refers to a set of competencies to process, understand, and reason with affective information. Recent studies suggest ability measures of experiential and strategic EI differentially predict performance on non-emotional and emotionally laden tasks. To explore cognitive processes underlying these abilities further, we varied the affective context of a traditional letter-based *n*-back working-memory task. In study 1, participants completed 0-, 2-, and 3-back tasks with flanking distractors that were either emotional (fearful or happy faces) or non-emotional (shapes or letters stimuli). Strategic EI, but not experiential EI, significantly influenced participants’ accuracy across all *n*-back levels, irrespective of flanker type. In Study 2, participants completed 1-, 2-, and 3-back levels. Experiential EI was positively associated with response times for emotional flankers at the 1-back level but not other levels or flanker types, suggesting those higher in experiential EI reacted slower on low-load trials with affective context. In Study 3, flankers were asynchronously presented either 300 ms or 1000 ms before probes. Results mirrored Study 1 for accuracy rates and Study 2 for response times. Our findings (a) provide experimental evidence for the distinctness of experiential and strategic EI and (b) suggest that each are related to different aspects of cognitive processes underlying working memory.

## 1. Introduction

Since the concept of emotional intelligence (EI) was introduced in the early 1990s, many experimental and correlational studies have been conducted to investigate its theoretical underpinnings and practical implications to human functioning, e.g., (Checa and Fernández-Berrocal 2015). Despite this wealth of studies, one aspect that remains unclear is the cognitive processes underlying this construct (Schneider et al. 2016). Contemporary researchers are starting to explore the relationships between ability EI and human cognition, specifically in relation to “hot” (emotional) versus “cold” (non-emotional) executive functions. They have yet to fully map out the equivalence (or isomorphism) of cognitive counterpart processes within the conceptual dimensions of ability EI. Given these theoretical gaps, a central goal of the current study was to explore ability EI’s relations to individual differences in working memory updating using the *n*-back task.

Working memory is an essential cognitive system in which incoming information is maintained and processed. The importance of working memory is that these processes occur despite interference or distraction (Miyake and Shah 1999). There are many tasks that purportedly measure working memory, but a common paradigm used by researchers is the *n*-back task. In a typical *n*-back task, participants are presented with a series of visual or verbal stimuli. For each stimulus presentation, they are asked whether the current one matches the stimulus previously seen *n* trials before. For example, in a 3-back letter task, participants have to decide whether the presented letter (e.g., “c”) is the same one presented in trial number *n*-3. The *n*-back task is considered a working memory task because it requires constant encoding of information, a temporary store for each stimulus presented, and a continuous processing or updating of each subsequent presentation. At the same time, the participant must inhibit irrelevant items, which must be removed from the memory store. Because of the cognitive subprocesses, the *n*-back task is also described to be measuring executive attention (Kane and Engle 2002). However, unlike a traditional letter-target *n*-back task, we embedded emotional and non-emotional distractors that flanked the centrally located target probes. The specifications for these distractors were varied across three studies so as to examine the contributions of ability EI to two key aspects of executive functions: emotional versus non-emotional inhibition and working memory updating.

### 1.1. Emotional Intelligence Abilities

A conceptual debate on the basis of EI has been ongoing for decades with several competing and complementary models being developed. The theoretical development of EI can be divided between two main perspectives: (1) trait EI and (2) ability EI (Mayer et al. 2008). From the trait perspective, EI is about one’s self-perceptions and dispositions. Such data are usually obtained via self-report measures. The commonality between the trait perspective and the self-report perspective is that both measure subjective perceptions of one’s emotional abilities. They typically involve self-report of emotional experiences and people’s behavioural preferences or styles relating to emotional expressions. A key differentiator of the trait EI perspective is that such measures do not correspond to the traditional paradigm for assessing general intelligence (*g*) or specific problem-solving abilities (e.g., fluid reasoning). 

More important to the present set of studies is the ability perspective of EI (Mayer et al. 2016). The ability EI perspective examines those capabilities specialized for the processing of emotional information. The main method of investigation are performance assessments of emotional-based competencies and abilities in which objective answers or optimal responses exist, as determined by an expert or general consensus. The Mayer-Salovey-Caruso Emotional Intelligence Test (MSCEIT; Mayer et al. 2002) is a prototypical test of ability EI. The ability model posits that EI is best viewed as an intelligence that is based on (or built upon) the abilities to use or reason about emotions. Mayer et al. (2016) suggests that ability EI focuses on hot information processing. Several researchers, notably Gutiérrez-Cobo et al. (2017a), have found correlations between participants’ performance on emotion-related tasks with ability EI but not on trait EI measures. In other words, EI consists of a class of abilities that involve processing emotional information and the effective utilizations of emotional information towards fulfilling the goal at hand. 

If ability EI pertains in the way just described, perhaps a logical step forward in its conceptualization is to examine its relationships and broader contributions to human cognition. Mayer et al. (1999) as well as Elfenbein and MacCann (2017) suggest that there are two key facets of ability EI: Experiential EI and Strategic EI. According to Mayer et al. (1999), the facet of Experiential EI essentially involves the abilities to identify or perceive the emotions of others. It is also considered to be reflective of lower-order abilities in emotional processing. On the other hand, Strategic EI describes the online emotional reasoning abilities and how emotions can be appropriately managed to suit the context at hand. It is also considered to be indicative of higher-level or conscious emotional information processing. Experiential EI and Strategic EI together form the binary aspects of EI as described by Mayer et al. (2003). Abilities under the Experiential EI facet are used for rapid perceptual processing of emotional information while Strategic EI abilities are used for deliberate (or strategic) reasoning and control of emotional information (Elfenbein and MacCann 2017). Importantly, Strategic EI involves the abilities to modulate or regulate emotions in oneself and manipulate others in the pursuit of one’s goals (Mayer and Salovey 1997). The two facets of Experiential and Strategic are central to the Mayer et al. (1999) conceptualization of ability EI, such that the MSCEIT provides two area scores that correspond to them, respectively. For example, the Experiential EI score is obtained from combining Branch 1: Perceive Emotions and Branch 2: Facilitating Thought whereas the Strategic EI score is obtained from combining Branch 3: Understanding Emotions and Branch 4: Managing Emotions. 

A recent study by Gutiérrez-Cobo et al. (2017a) examined the relationships between EI and hot/cold executive functions (i.e., cognitive control). In their study, participants completed measures of EI according to either the self-report ability model (Trait Meta-Mood Scale, TMMS), self-report mixed model (Emotional Quotient Inventory—Short Form, EQi:S), or performance-based ability model (MSCEIT). The researchers also had participants complete both hot and cold variants of an executive function task (i.e., go/no-go task). For the hot variant, participants had to press a button (“go” trials) for a target emotional face and to refrain (“no-go” trials) for a neutral face, and vice versa in different blocks. For the cold variant, participants completed a go/no-go task using shapes and colours as stimuli. The researchers found that, overall, positive correlations (in the order of *r* = 0.15 to *r* = 0.18) between participants’ scores on the managing branch on the MSCEIT and their reaction times when “go” stimulus was an emotional face but not with neutral faces. Scores on the MSCEIT’s managing emotion branch were also negatively associated with false alarm rates for happy and angry faces (in the order of *r* = −0.15 to *r* = −0.19). While these associations were small, these findings suggest that Strategic EI abilities, which entail the managing branch, could contribute positively to one’s executive control for emotional conflict. 

In a follow-up study, Gutiérrez-Cobo et al. (2017b) examined whether EI was linked to working memory capacity (WMC) and whether this relationship was different if the WMC task was of a hot (affective) or cold variant. They had participants complete a hot *n*-back task using angry, happy, and neutral face stimuli (e.g., “Was this happy face the same happy face *n*-steps ago?”), as well as to complete a cold *n*-back task (e.g., whether the presented letter is the same as the one *n*-steps ago). Once again, they found that the managing branch of EI was positively associated with performance on the hot (or emotional face) *n*-back task but not on the cold variant (i.e., updating letters). This finding was intriguing and held conceptual implications to the broader emotional regulation literature, as previous researchers (e.g., Schmeichel and Demaree 2010) found that WMC could be related to one’s ability to regulate emotions. In Gutiérrez-Cobo’s et al. study, WMC was implicated in the managing branch of EI. This suggested that the ability to regulate emotions both in self and in others (the definition of the Strategic EI facet on the MSCEIT) requires the cognitive control of emotional information. These findings are also consistent with other studies that demonstrated emotional working memory training could improve emotion regulation (Schweizer et al. 2013). In other words, Strategic EI abilities are utilized when the cognitive task requires a degree of emotional control and inhibition. 

One important limitation of Gutiérrez-Cobo et al. (2017a, 2017b) is that their studies did not specify the locus of the EI contributions to the performance on the cognitive tasks. In their study using a hot and cold *n*-back task (Gutiérrez-Cobo et al. 2017b), the targets were either emotional faces (hot variant) or consonant letters (cold variant). As such, it was uncertain whether the implicated EI processes were in relation to: (1) affective processing (i.e., simple updating of emotional information) or (2) affective inhibition (i.e., the ability to inhibit an emotional context when performing on the task). Another minor limitation is that Gutiérrez-Cobo et al. (2017b) did not employ a more standardized way of calculating *n*-back performance. For example, they calculated *n*-back accuracy based on the total number of hits instead of the more commonly used indices of percentage (or proportion) of hits divided by the number of trials (i.e., as used in signal detection theory; Jacola et al. 2014; Kane et al. 2007). They also calculated “miss rate” as the total number of “no” responses to *n*-back targets. Additionally, Gutiérrez-Cobo et al. (2017b) used a two-key response version of the *n*-back task, but they did not take the rate of correct rejections (or false alarms) into consideration. Correct rejection rates are a useful metric for calculating *n*-back accuracy (Jacola et al. 2014). Taken together, it was not possible to determine whether the contributions of ability EI were due to the challenges in inhibiting responses to a non-target probe or due to failures when responding to a target. In our studies, we also employed a two-key administration to ensure separation of participants’ responses but also utilized participants’ hit rates and correct rejection rates to obtain a better estimate for emotional *n*-back performances. 

Another limitation of Gutiérrez-Cobo’s et al. (2017b) study is that they did not find significant correlations between response latencies for correct (or hit) trials on the *n*-back task with any of their EI measures, including the MSCEIT. We find this non-relationship to be surprising because performance on the *n*-back task is not solely restricted to accuracy rates. Importantly, response latencies have previously been found to predict unique individual differences in relation to intelligence. For example, Hockey and Geffen (2004) found stable and positive correlations between cognitive abilities and correct response times on an *n*-back task. Similarly, Gevins and Smith (2000) found that participants in a high cognitive ability group (as measured using the WAIS-R) were significantly faster on the *n*-back task when compared with low-ability participants. 

In part to answer these limitations, as well as to explore the relationships between EI and working memory in greater detail, the *n*-back task used in the current study held the type of target stimuli constant (letters only) while varying the affective context demands of the task. We created a task called the Emotional Flanker N-back (EFNB), which was modified from the original Emotional Face N-back task (EFNBACK; Ladouceur et al. 2009). In the original EFNBACK, the task consisted of presenting a standard letter *n*-back paradigm wherein each trial was either flanked by emotional faces (happy, fearful, neutral) or no picture. In our EFNB task, we kept the happy and fearful faces but changed the neutral/no-picture conditions. Instead of using neutral faces or no faces for the cold task variant, we used: (1) geometric shapes and (2) letters for the flanking distractors. The purpose of using shape flankers and letter flankers was to determine whether there was a difference when responding in the presence of affective or non-affective flankers within the same experimental paradigm and whether this difference was related to participants’ EI abilities. 

Another difference from the original EFNBACK task was that we included a third memory-load condition (Study 1). In the original task, there were only two memory-load conditions (0-back, 2-back). There were two reasons for adding a third memory load (3-back) into the EFNB task. First, for any increase in *N*, there is an increasing demand in processing load. As load increases, participants have to shift or expand the attentional focus between the presented stimuli for comparison in working memory (McElree 2001) as well as discriminate and inhibit stimuli that no longer need to be maintained in working memory (Kane et al. 2007). In the context of our current study, we hypothesized that EI abilities would be implicated when inhibiting distracting emotional information and that this would be more readily observed as processing load increased. Second, EI-related differences might be more pronounced in the hot *n*-back task as the demands on WMC increased. If EI is considered to be a measurable mental ability (Mayer and Salovey 1997), then increasing the demands of an EI-implicated task should lead to further differences in participants’ performances on an emotional *n*-back task. Such a proposal would also be congruent with MacCann et al. (2014), who argued for EI as a broad intelligence that could possibly impact cognitive performance. It would also be congruent with Mayer’s et al. (2016) updates to their original ability EI model; specifically, EI is a class of broad intelligence that is utilized when working on “hot” (or emotional) information (p. 3). Finally, the 3-back task also requires a considerable amount of mental effort and is cognitively challenging at this difficulty level (Jaeggi et al. 2010). A person with higher EI abilities may be much better in managing the affective distractors and memory load demands as compared to a person with lower EI abilities. 

Finally, we used emotional faces as flanking distractors for two reasons. First, faces are meaningful sources of information that receive preferential processing and bias the allocation of one’s attention to themselves (Eimer and Holmes 2002; Fenske and Eastwood 2003). Additionally, emotional stimuli—especially negative ones—are likely to be processed automatically and rapidly by the brain (i.e., through the amygdala, e.g., Diano et al. 2016). Emotional face flankers capture one’s limited resources for information processing, thereby impairing performances on tasks measuring working memory (Dolcos et al. 2008). Second, the use of face flankers allows for the comparisons of affective states in their impact on memory updating (e.g., between happy faces and fearful faces flankers). In the EFNB task, the faces flankers are to be ignored while working on the *n*-back task. However, negative faces capture attention nonetheless, even when people are explicitly instructed to ignore them (Eastwood et al. 2003). As such, negative face flankers are likely to disrupt *n*-back performance more so than positive face flankers and by extension, the letter or shape flankers. However, higher EI individuals are expected to be less susceptible to this disruption because they are able to allocate more resources towards inhibiting emotional distractors (Checa and Fernández-Berrocal 2015).

### 1.2. Purpose of Study 1

Study 1 had two main objectives. First, it aimed to explore the notion that EI abilities are differentially related to performance in a hot and cold working memory updating task. To this end, we utilized flanker distractors that were either affective (happy faces or fearful faces) or non-affective (letters and shapes) and embedded them in a standard letter *n*-back task. Second, we were interested to examine whether varying the memory load (0-back, 2-back, and 3-back) would have an impact on participants’ performances on the hot/cold variants and whether these were influenced by their EI. The 0-back load condition was akin to a monitoring task (e.g., go/no-go) and primarily taps inhibition control, while the 2-back and 3-back load conditions tap both inhibition and updating processes. 

There were two sets of core hypotheses of interest: four relating to the EFNB task and a set of moderation hypotheses related to the EI abilities. First, we hypothesized that an increase in updating load would impede *n*-back performance (i.e., reduced accuracy rates and increased reaction times) in both affective and non-affective flankers, thereby replicating the standard *n*-back memory load effect. Second, we expected that performance would be worse for the affective flankers because emotional faces capture attention more readily than non-facial features (Eastwood et al. 2003), thereby impeding *n*-back performances in the affective flanker conditions even further vis-à-vis neutral flanker conditions. In other words, we hypothesized that *n*-back performance would be significantly poorer for both the happy face and fearful face flankers conditions when compared to neutral flankers (letters and shapes). A third and related hypothesis was that *n*-back performance would be poorer for the fearful flankers condition as compared to the happy flankers condition because negative emotions are thought to be more attention-grabbing than positive ones (Hodsoll et al. 2011). Finally, we expected that *n*-back performance would be least impacted by shape flankers when compared to letter flankers because the probes for our *n*-back task were letters. As such, letter flankers were expected to generate more interference because they shared features with the probes (Baylis and Driver 1992). 

For specific EI-related hypotheses, we first hypothesized that *n*-back performance on the hot 2-back and 3-back conditions would be significantly moderated by participants’ Strategic EI score on the MESCIT, as suggested by the positive correlations found in Gutiérrez-Cobo’s et al. (2017b) study. We also expected that EI abilities would not be significantly related to *n*-back performance when the flanking distractors were either neutral shapes or letters. 

In summary, we manipulated the *n*-back memory load and the valence of flanking distractors to critically test three interference effects: (1) the interference of affective faces on working memory performance as compared to neutral flankers; (2) the interference of happy face flankers versus fearful face flankers; and (3) the contributions (moderation) of Experiential EI ability and Strategic EI ability with specific regard to these interferences. The affective face distractors would reduce available cognitive resources allocated to working memory, but this impairment was expected to be less pronounced in participants with higher Strategic EI abilities. If EI was indeed an ability to cope with one’s own emotions in an adaptive manner, it would mostly likely have been implicated only when the cognitive task required inhibition of emotional information. This was in line with Mayer and Salovey’s (1997) notion that EI abilities are about accurately facilitating and inhibiting emotional signals in response to a situation. In our *n*-back task, the focus was evidently more on the inhibition of emotional interference arising from the signals generated from emotional face distractors. 

## 2. Materials and Methods

### 2.1. Participants and Power Analysis

A total of 85 (66 females, mean age = 20.54) undergraduate students from the University of Sydney participated in the study as part of their course credit requirement. Initially, 89 participants had completed both the MSCEIT and the *n*-back task. However, 4 participants’ data were dropped from the analysis due to a technical issue with the testing computer (i.e., a number of responses were not recorded in multiple blocks). The selection criteria were as such: (1) be above 18 years old in age, (2) moderately proficient in English, and (3) no colour blindness. Power analysis was conducted using MorePower 6.0 (Campbell and Thompson 2012) with the desired power of 0.80 and a medium effect size 0.06 (η^2^). We also used PASS 15 Power Analysis and Sample size software (PASS 15 2017) to calculate for the inclusion of covariates, with the specifications of 3 covariates, *R^2^* = 0.30, and the number of group means being compared = 15. The required sample size was 60. Ethics approval was obtained from the Human Research Ethics Committee, University of Sydney.

### 2.2. Design and Procedure

A fully within-subjects design was used. Participants completed all study tasks in a single session lasting approximately 90 min. The MSCEIT was always administered first to participants and then followed by the computerized *n*-back task. Participants completed the tasks in groups of up to 10 in computer labs at the University of Sydney. 

### 2.3. Mayer-Salovey-Caruso Emotional Intelligence Test (MSCEIT)

The MSCEIT is comprised of eight subtests and assesses how well an individual solves emotion-laden problems across four branches of EI as delineated by Mayer and Salovey (1997): (B1) Perceiving Emotions, (B2) Facilitating, (B3) Understanding Emotion, and (B4) Managing Emotions. There is a total of 141 test items in the MSCEIT, and the test has been validated for adults aged 17 years and older. For each item, participants were instructed to indicate effectiveness from a list of options, ranging from 1 = very ineffective to 5 = very effective, or to the presented emotions, ranging from 1 = not at all present to 5 = present to a great extent. Expert norms were used for score derivations. In the current study, we focused mainly on the two area scores: (1) Experiential EI; and (2) Strategic EI. The MSCEIT was administered and scored automatically using the online MHS (Multi-Health Systems Inc.) portal. 

### 2.4. Emotional Flanker N-back

**Figure 1:**
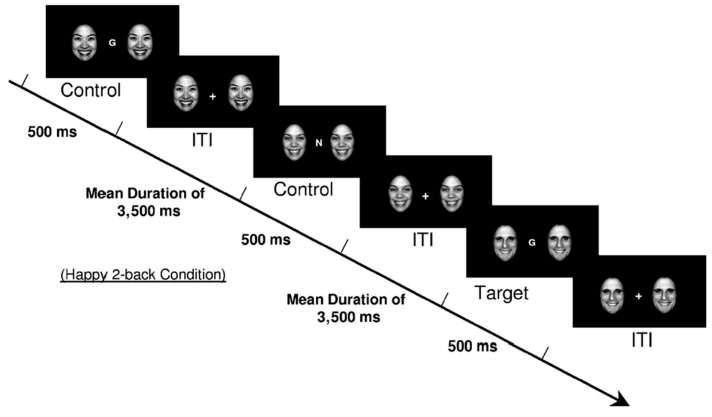
Illustration of the original Emotional Face N-back task (Ladouceur et al. 2009). This is an example of the happy-face 2-back memory-load condition. ITI = intertrial interval. Printed with permission.

The Emotional Flanker N-back (EFNB) task used in Study 1 was adapted from the original face *n*-back task, as described in Ladouceur et al. (2009). The *n*-back component itself consists of visually presenting a series of letters (trials) on the centre of a screen. Letter consonants were used as target and non-target probes in the ratio of 1:2, respectively. Participants were required to indicate whether or not the letter probe on the screen was the same as the letter presented *n* trials previously. Unlike Ladouceur’s et al. version, we used a 2-key version which required participants to respond to every trial. Participants pressed either the letters “a” for “match” or “l” for “no match” to indicate whether they believed the current letter was identical to the letter presented *n* trials ago. Figure 1 presents an example of a 2-back happy face flankers condition as used by Ladouceur et al. (2009).

For the 0-back, 2-back, and 3-back memory load conditions, there were four possible flanker distractor conditions (happy faces, fearful faces, geometric shapes, and letters). Instead of using neutral faces or “no-picture” conditions as control (i.e., as in the original version by Ladouceur et al. 2009), we used shapes and letters as distractors to control for the interference related to an emotional distractor on either side of the target letter probes in all the memory load conditions. The geometric shape and letter distractors allowed the comparisons regarding the interference effects of emotional stimuli against interference effects of affectively neutral stimuli. We did not include a no-distractor control condition because it would not have been possible to generate any meaningful interference to the *n*-back task if no distractors were present. Finally, we did not use neutral faces as a control because such stimuli had been found to be conveying negative affect by young and old participants (e.g., negativity basis; Tottenham et al. 2013). 

The EFNB task entailed two complete repetition runs of the 12 blocks created by crossing the three memory-load conditions (0-back, 2-back, and 3-back, all with letter stimuli) and four flanking distractor conditions (happy faces, fearful faces, geometric shapes, and letters). The 12 blocks within each repetition run were presented in a different random sequence for each participant. Each block consisted of 12 trials, with letter probes centrally presented and flanked on both sides by stimuli for the respective condition. Each trial presented the letter probes for 500 ms, and a fixation cross replaced the probes during the intertrial interval. Each letter probe was drawn from a pool of 20 consonants. This interval had a mean of approximately 3500 ms (randomly determined from a range of 3000 ms to 4000 ms, in 11 discrete 100 ms lots). The response window for participants was set at max 3000 ms. The response window in which participants could record a response lasted until 3000 ms after the presentation of the letter probe (fixation cross). Flankers were presented throughout the trial, with identical flankers presented on the right and left sides of the letter probes in any given trial. 

The face flanker stimuli were pictures of actors (males and females) displaying fearful- and happy-face (mouth open) expressions. These pictures were taken from the NimStim set available at www.macbrain.org (Tottenham et al. 2009), with 10 females (numbers: 2, 5, 6, 7, 8, 9, 13, 14, 18, and 19) and 10 males (numbers: 21, 23, 24, 27, 29, 30, 33, 37, 41, and 42). All images were 800 × 600 pixels and cropped into an oval shape. The modified pictures were then aligned so that all actors’ faces and eyes appeared at the same location across trials. Letter flankers were drawn from a pool of vowels (“A”, “E”, “I”, “O”, “U”) while the shapes flankers were geometric shapes previously used by Jaeggi et al. (2010). Participants responded as quickly as possible with their index fingers to the target letter probes. Brief instructions were presented on the screen at the beginning of each block to inform participants which memory load condition they were doing. They also completed a practice block of nine trials each for 0-back, 1-back, 2-back, and 3-back, with end-of-block performance feedback. No feedback was provided in the actual test blocks. The EFNB task was coded and administered using the software Inquisit 5 (2016). 

### 2.5. Results

Responses outside the response window were considered as “No Response” and removed; these amounted to fewer than 1% of all responses. We calculated the accuracy rates for the *n*-back task across all flanker-type conditions and memory load conditions, where accuracy rates = (hits + correct rejections)/total actual trials for each block (i.e., the first *n* trials are not counted because they do not have a meaningful response). In other words, there were *n* + 12 trials per experimental block. Means (RTs) for hit trials and correct rejection trials were calculated and log-transformed. Because of the separation of RTs for hit and correct rejections trials, the number of participants for RT analyses could be lower than the total number of participants. 

Following Ladouceur et al. (2009), a series of repeated-measures MANCOVAs were performed on participants’ accuracy rates and reaction times for all correct trials. The covariates entered into the MANCOVAs were the two MSCEIT’s area scores: (a) Experiential EI; and (b) Strategic EI. Where appropriate, the test statistic reported is Wilks’ lambda. Additionally, Greenhouse–Geisser and Bonferroni corrections were used as indicated. Table 1 presents the correlations between participants’ MSCEIT scores (mean-centered) for Study 1. As expected, scores between each branch of the MSCEIT were significantly associated with each other. 

#### 2.5.1. Accuracy

The analyses revealed main effects of Memory Load, *F*(2, 81) = 96.936, partial-η^2^ = 0.705, Wilks’ Λ = 0.295, *p* < 0.001, but no main effects of Flanker Type, *F*(3, 80) = 1.689, partial-η^2^ = 0.060, Wilks’ Λ = 0.940, *p* = 0.176. Importantly, the Memory Load × Flanker Type interaction was significant, *F*(6, 77) = 2.481, partial-η^2^ = 0.162, Wilks’ Λ = 0.838, *p* = 0.030 (see Table 2 for means and SDs for each condition).

As expected, accuracy rates decreased significantly as memory load of the *n*-back task increased. According to pairwise comparisons with Bonferroni adjustments, participants at the 2-back fearful face flanker condition had significantly lower *n*-back accuracy when compared with shape flankers (*p* = 0.005). The difference between the 2-back happy face flankers condition and shape flankers condition reached near significance (*p* = 0.067). All other comparisons were not significant. Therefore, the interference effects of the emotional face flankers were most evident at the medium memory load (2-back) and not when memory load was high (3-back) or none (0-back). 

Importantly, the covariate Strategic EI significantly interacted with Memory Load, *F*(2, 81) = 4.743, partial-η^2^ = 0.105, Wilks’ Λ = 0.895, *p* = 0.011. To aid in the interpretation regarding the interactions between Memory Load and Strategic EI, we split the covariate into three bins based on even percentiles for all participants. Instead of performing a simple median split, the more groups a variable has, the better it is able to replicate the patterns found in the original variable (DeCoster et al. 2011)^1^. We named the three groups: Low Strategic EI (*M* = 80.16, *SD* = 7.06), Mid Strategic EI (*M* = 95.58, *SD* = 5.33), and High Strategic EI (*M* = 110.99, *SD* = 8.88). Descriptive statistics for the groups and the *n*-back accuracy rates at each memory load condition are reported in Table 3. 

**Figure 2:**
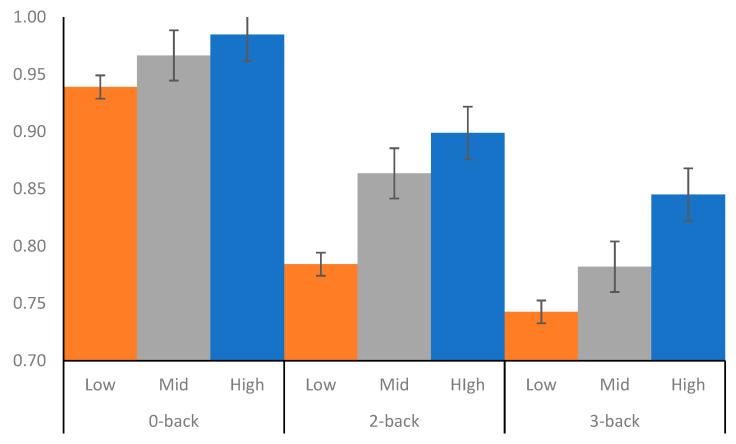
Accuracy According to Emotional Reasoning Group in Study 1; Note: Error bars are standard errors of the mean.

Pairwise comparisons between overall accuracy for 0-back, 2-back, and 3-back load conditions and the three Strategic EI groups were conducted with Bonferroni adjustments (see Figure 2). At the 0-back task, participants in the Low Strategic EI group had significantly lower accuracy rates (*p* = 0.002) than the High Strategic EI group but were marginally significant with the Mid Strategic group (*p* = 0.054). There were no significant differences between the Mid Strategic group and the High Strategic group (*p* = 0.656). At the 2-back condition, the Low Strategic EI group had significantly lower accuracy rates than the High Strategic EI group (*p* < 0.001) and Mid Strategic EI group (*p* = 0.013). There were no significant differences between the Mid Strategic EI group and the High Strategic EI group (*p* = 0.259). 

At the 3-back condition, the Low Strategic EI group had significantly lower accuracy rates than the High (*p* = 0.002), but not Mid Strategic EI group (*p* = 0.224). However, the difference between the Mid and High Strategic EI group was marginally significant (*p* = 0.054). The pattern of results indicates that participants with higher Strategic EI were significantly more accurate on the *n*-back task as compared to participants with lower Strategic EI, and this performance enhancement was the evident at all memory load conditions.

#### 2.5.2. Reaction Times for Hits

Descriptive statistics for correct RTs (before log-transformed) are reported in Table 4 (hit trials). Two-way repeated measures MANCOVAs were performed in order to test whether flanker type had differential effects on correct RTs (log-transformed) depending on memory load. As expected, RTs for hit trials increased with increases in *n*-back load. Analyses yielded significant main effects of memory load, *F*(2, 79) = 82.433, partial-η^2^ = 0.676, Wilks’ Λ = 0.324, *p* < 0.001, and flanker types, *F*(3, 78) = 4.685, partial-η^2^ = 0.153, Wilks’ Λ = 0.847, *p* = 0.005. There was no significant interaction effect of memory load and flanker type, *F*(6, 75) = 1.014, partial-η^2^ = 0.075, Wilks’ Λ = 0.925, *p* = 0.423. Additionally, we also found no significant interaction effects for either Strategic EI or Experiential EI as covariates.

Using post hoc pairwise comparisons, the difference between each pair of memory loads was significant (*p* < 0.001). RTs for hit trials on the *n*-back task were the longest for 3-back tasks, shorter on 2-back, and the shortest for 0-back tasks, regardless of the flanker type. Regarding main effects of flanker type, pairwise comparisons revealed statistically significant differences between fearful faces and letters (*p* = 0.020) and between letters and shapes (*p* = 0.029). All other differences were not significant (*p* > 0.05). Therefore, RTs for hits were the longest for letters and happy faces, but it was significantly shorter for fearful faces than for letters. Hit RTs for shape flankers were the lowest and significantly lower than for letter flankers. Table 5 presents estimates of correct reaction time for each flanker type (fearful faces, happy faces, letters and shapes) collapsed across all memory load conditions. 

#### 2.5.3. Reaction Times for Correct Rejections 

A similar pattern emerged for the RTs for correct rejection trials. Main effects of *n*-back memory load, *F*(2, 80) = 73.66, partial-η^2^ = 0.648, Wilks’ Λ = 0.352, *p* < 0.001, and flanker types *F*(3, 79) = 7.228, partial-η^2^ = 0.215, Wilks’ Λ = 0.785, *p* < 0.001 were significant. There was no significant interaction effect of memory load and flanker type *F*(6, 76) = 0.522, partial-η^2^ = 0.040, Wilks’ Λ = 0.960, *p* = 0.790 (see Table 6). 

Using pairwise comparisons, the difference between each pair of memory loads was significant (*p* < 0.001). RTs for correct rejections were the longest for 3-back tasks, shorter on 2-back, and the shortest for 0-back tasks regardless of the flanker type. Regarding main effects of flanker type, pairwise comparisons revealed a statistically significant difference between fearful faces and letters (*p* < 0.001), and between letters and shapes (*p* = 0.004). All other differences were non-significant. Table 7 presents estimates of RTs for correct rejection for each flanker type collapsed across all memory load conditions. 

Interestingly, we found a significant interaction effect for Memory Load x Experiential EI, *F*(2, 80) = 3.503, partial-η^2^ = 0.081, Wilks’ Λ = 0.919, *p* = 0.035. The interaction term for Flanker x Strategic EI also reached near significance *F*(3, 79) = 2.641, partial-η^2^ = 0.091, Wilks’ Λ = 0.909, *p* = 0.055. None of the correlational pairs between Experiential EI and the correct RTs were significant when RTs were aggregated within each load condition. 

### 2.6. Discussion for Study 1

When considering *n*-back performance without any EI influences, the results indicate impaired *n*-back accuracy rates when participants were presented with fearful face flankers, but this was only evident at the 2-back condition. Importantly, we found that Strategic EI, but not Experiential EI, significantly contributed to the *n*-back performance as memory load increased although this was irrespective of flanker types. This was contrary to the previous findings by Gutiérrez-Cobo et al. (2017a, 2017b) who found that only Branch 4: Managing Emotion (i.e., one component of Strategic EI) was associated with better performance in cognitive tasks involving emotional stimuli. 

In the context of our present study, it seemed that Strategic EI contributed to the overall accuracy performance of a cognitively challenging task such as the *n*-back. We also found a significant interaction between Experiential EI and memory load for correct rejection RTs. None of the correlational pairs between Experiential EI and RTs rates were significant. Nevertheless, this result was interesting because it suggested that Strategic EI and Experiential EI have differential contributions to performance in the *n*-back task (i.e., a working-memory updating task). Specifically, Strategic EI might be more related to accuracy while Experiential EI could be more related to the speed of processing. 

## 3. Study 2

Study 1 demonstrated that Strategic EI significantly enhanced participants’ performance when memory load demands were varied from low (0-back) to high (3-back). We were keen to explore why Strategic EI’s contributions were not dependent on the flanker type despite the presence of an emotional interference effect of fearful face flankers in the *n*-back task. We were also intrigued by the possibility that Experiential EI could contribute towards RTs performance. To explore these questions, we conducted two additional studies where various parameters of the EFNB task were further modified. These changes were done to examine in more detail the contributions of ability EI to participants’ updating performance while having to inhibit emotional distractors simultaneously. Studies 2 and 3 also served the purpose of replicating the effects of ability EI on the *n*-back task. 

### 3.1. Method

#### 3.1.1. Participants and Power Analysis

Sixty-one participants (34 females, mean age = 21.38 years) from the online research platform Prolific (www.prolifico) were recruited^2^. We removed one participant due to unusually low accuracy rates on the *n*-back task. The selection criteria were as such: (1) be between 18 years and 30 years old in age, (2) have good proficiency in English, (3) be currently studying at an undergraduate university program, and (4) have no colour blindness. We set these criteria to recruit participants that closely mirrored those who had completed Study 1. Power analysis was conducted using MorePower 6.0 and PASS 15. The required sample size was 32, with the desired power of 0.80 and an effect size of 0.14. The sample size requirements increased to 48 to account for the inclusion of the EI covariates, with the specifications of 3 covariates, *R*^2^ = 0.30, and the number of group means being compared = 12. Ethics approval for online research participation was obtained from the Human Research Ethics Committee, University of Sydney.

#### 3.1.2. Design, Materials, and Procedure

The design and protocols were largely similar to those employed in Study 1, but with several changes made to the number of trials, load condition, and flanker type. The EFNB task used in Study 2 had three memory load conditions: 1-back, 2-back, and 3-back load conditions as well as 3 different flanker types: fearful faces, happy faces, and geometric shapes. Unlike in Study 1, we removed the letter flanker condition because it seemed to generate distractor interferences that were not unique to the emotional/non-emotional dimensions of this task. Rather, the interferences of letter flankers were generated because of the congruent versus incongruent nature of the letter flankers in relation to the *n*-back probes (e.g., letter flankers were more congruent to the target probes as compared to the shape flankers). We also dropped the 0-back condition as participants’ accuracy was close to 1.0 for all conditions in that memory load condition. Instead, we used a 1-back task variant (low memory load), while still having participants complete the 2-back and 3-back levels.

Finally, to make the *n*-back task more sensitive to emotional interferences, we substantially increased the number of trials within each condition. As such, participants were only required to complete a single run for each load and flanker condition instead of the experimental blocks being repeated three times (as in Study 1). The number of actual trials per block was 30, with a ratio of 1:2 for targets and non-targets. Participants completed a total of 270 actual trials over 9 blocks. The duration for Study 2 was approximately 60 min. Participants completed the *n*-back task first. Subsequently, they were then invited to complete the MSCEIT using a separate online study link. 

### 3.2. Results

The scoring and analyses used were identical to Study 1, with Experiential EI and Strategic EI entered as covariates into the MANCOVAs. The only difference was the number of actual trials used to calculate accuracy. Table 8 presents the correlations between participants’ MSCEIT scores. Unlike Study 1, the relationships between the branches of the MSCEIT in Study 2 were not all significantly correlated. Branch 1: Perceiving Emotions was not significantly correlated with Branch 3: Understanding Emotions. Branch 2: Using Emotions was not significantly correlated with Branch 3: Understanding Emotions and Branch 4: Managing Emotions. 

#### 3.2.1. Accuracy

The MANCOVA revealed main effects of memory load, *F*(2, 56) = 104.398, partial-η^2^ = 0.789, Wilks’ Λ = 0.211, *p* < 0.001, but no main effects of flanker type, *F*(2, 56) = 1.148, partial-η^2^ = 0.039, Wilks’ Λ = 0.961, *p* = 0.325. The Memory Load × Flanker Type interaction was significant, *F*(4, 54) = 2.797, partial-η^2^ = 0.172, Wilks’ Λ = 0.828, *p* = 0.035. According to pairwise comparisons with Bonferroni adjustment, accuracy was significantly lower (*p* = 0.012) for fearful face flankers when compared to shape flankers at the 2-back load condition. All other comparisons were not significant. This indicates an emotional (fearful) interference effect for accuracy rates at the 2-back level but not at the 1-back or 3-back conditions. Table 9 displays estimated marginal means of accuracy for each combination of categories of memory load and flanker type.

Unlike Study 1, there were no significant interaction effects of Memory Load x Strategic EI, *F*(2, 56) = 0.912, partial-η^2^ = 0.032, Wilks’ Λ = 0.912, *p* = 0.408. All other interactions with the two covariates were also not significant.

#### 3.2.2. Reaction Times for Hits

The main effects of *n*-back load, *F*(2, 54) = 46.288, partial-η^2^ = 0.632, Wilks’ Λ = 0.368, *p* < 0.001, were significant but not for flanker type, *F*(2, 54) = 1.225, partial-η^2^ = 0.043, Wilks’ Λ = 0.957, *p* = 0.302. There was a significant interaction effect of *n*-back load and flanker type, *F*(4, 52) = 3.548, partial-η^2^ = 0.214, Wilks’ Λ = 0.786, *p* = 0.012. Using post hoc pairwise comparisons, the differences between each pair of memory loads were significant (*p* < 0.001), except between the 2-back and 3-back conditions (*p* = 0.061).

At the 3-back level, hit RTs for fearful face flankers were significantly lower as compared to shape flankers (*p* = 0.035). The hit RTs for happy face flanker were also significantly lower as compared to shape flankers (*p* = 0.022). This was an unexpected finding as it indicates that emotional flankers facilitated response latencies for hit trials at the 3-back level. In other words, the results are suggestive of an emotional facilitation effect at high memory load (see Table 10). 

Unlike Study 1, we found a near significant interaction effect for Memory Load x Experiential EI, *F*(2, 54) = 3.036, partial-η^2^ = 0.101, Wilks’ Λ = 0.899, *p* = 0.056. To explore this near-significant interaction, correlational analyses indicated that Experiential EI was significantly and positively associated at the overall 1-back level, *r*(59) = 0.330, *p* = 0.011. Within the 1-back level, Experiential EI was positively associated with hit RTs when presented with fearful face flankers, *r*(60) = 0.290, *p* = 0.024, and with happy face flankers, *r*(60) = 0.281, *p* = 0.030. It was also positively correlated at trials with shape flankers, *r*(59) = 0.325, *p* = 0.012. The pattern of results is intriguing as it suggests that as Experiential EI increased, response times on the 1-back task were longer as well.

#### 3.2.3. Reaction Times for Correct Rejections

The MANCOVAs revealed main effects of *n*-back load, *F*(2, 56) = 36.979, partial-η^2^ = 0.569, Wilks’ Λ = 0.431, *p* < 0.001, but not for flanker types, *F*(2, 56) = 0.394, partial-η^2^ = 0.014, Wilks’ Λ = 0.986, *p* = 0.677. There were no significant interaction effects of *n*-back load and flanker type, *F*(4, 54) = 0.888, partial-η^2^ = 0.062, Wilks’ Λ = 0.938, *p* = 0.478. The pairwise comparisons (see Table 11) reveal that the differences between each pair of memory loads were significant (*p* < 0.001), with the exception of 2-back and 3-back (*p* ~ 1.00).

Similar to Study 1, we found significant interactions with Load x Experiential EI, *F*(2, 56) = 3.218, partial-η^2^ = 0.103, Wilks’ Λ = 0.897, *p* = 0.048. Additionally, we found a significant Load × Flanker × Experiential EI interaction, *F*(4, 54) = 2.702, partial-η^2^ = 0.167, Wilks’ Λ = 0.833, *p* = 0.040. To determine the source of this 3-way interaction term, correlational analyses indicated that Experiential EI was significantly and positively correlated with correct rejection RTs at the 1-back level for fearful face flankers, *r*(60) = 0.295, *p* = 0.022, and for happy face flankers, *r*(60) = 0.265, *p* = 041. This pattern of results is similar to the analyses for the RTs for hit trials, but with the absence of the relationship between Experiential EI and neutral shape flankers. This suggests that an increase in Experiential EI led to slower RTs for correct rejection trials with emotional face flankers. 

### 3.3. Discussion for Study 2

The results for Study 2 indicate impaired accuracy rates for fearful faces and this was mainly observed at the 2-back condition. This indicates an emotional interference effect for fearful face flankers at the 2-back level, which replicated the same effect from Study 1. Interestingly, we found that response latencies were significantly faster for emotional flankers at the 3-back condition. Instead of an interference effect at the 3-back level, emotional face flankers prompted participants to respond quicker. As these response latencies were for correct trials only, this is suggestive of an emotional facilitation effect at the high memory load condition. 

In contrast to the findings of Strategic EI’s contributions in Study 1, we found that it was Experiential EI that were significantly related to performance (response latencies only) on the emotional *n*-back task. The associations between Experiential EI and RTs were positive, indicating that participants with higher experiential EI took significantly longer to respond correctly. The same relationship was not observed for neutral shape flankers. Our findings could be similar to Gutiérrez-Cobo’s et al. (2017a) study where they found that one aspect of Strategic EI (i.e., Branch 4: Managing Emotions) were positively correlated with RTs in an emotional go/no-go task. This positive relationship for RTs was observed when the go stimuli were either angry or sad faces (i.e., when the no-go stimuli were neutral faces), as well as when the no-go stimuli were angry, fearful, happy, or sad faces. 

In our study, we however found that it was Experiential EI that was associated with slower response times. One reason for this difference could be due to the type of cognitive task used in these studies. Because our *n*-back task utilized emotional face flankers, it was likely that participants with higher emotional perception abilities were more likely to encode and process these flankers. As such, these participants were more likely to require additional time to resolve the emotional interference arising from these face flankers. Nevertheless, our findings complement Gutiérrez-Cobo’s et al. (2017b). While they only found a positive relationship between the Managing Emotion branch and hit rates in an emotional *n*-back task, we found that Experiential EI was implicated in reaction times for trials in which participants correctly rejected the non-target *n-*back probes^3^. 

Despite the differences, both Gutiérrez-Cobo’s et al. (2017a, 2017b) findings and our results are similar to Austin’s (2009) study, where she found that participants with higher ability EI responded much more slowly to difficult test items as compared to participants with lower ability EI. Combining the results of Study 1 and Study 2 together, it appears that ability EI contributions to the *n*-back task differed depending on whether performances were measured using accuracy rates or response latencies. 

## 4. Study 3

We were surprised to find that Study 2 did not replicate the contributions of Strategic EI to the EFNB task. Instead, it was participants’ Experiential EI that significantly impacted performance on the *n*-back task. One possible explanation is that the effects of ability EI are not stable and may be dependent on the specific parameters of the cognitive task used in the research study. In contrast to previous published studies on ability EI and *n*-back performance, we had attempted to replicate the contributions of ability EI to working memory, updating Study 2. It was also surprising that RT performance at the 3-back level was facilitated by the emotional face flankers. To further explore whether we could elicit an interference effect (for accuracy) or facilitation effect (for RTs) at the 3-back level, we varied the temporal dynamics of distractor presentation during the emotional *n*-back task (i.e., the stimulus onset asynchrony (SOA) between the emotional distractors and the *n*-back probes). Emotional faces attract attention almost automatically (Eastwood et al. 2003). However, many models of cognitive control suggest that briefly presented emotional distractors can impair subsequent cognitive processing, similar to an emotional-induced attentional blink (McHugo et al. 2013). By having the emotional face distractors appear first before the *n*-back probes might induce a stronger emotional effect as attention would be drawn automatically to the emotional faces. Additionally, we also increased the number of flankers to 4 to surround the *n*-back probes on all sides (i.e., top, down, left, and right). Evidently, the task complexity of the emotional *n*-back task increased as well. As such, it was hypothesized that Strategic EI would play a more significant role in modulating participants’ performance.

### 4.1. Method

#### 4.1.1. Participants and Power Analysis

Sixty-three undergraduate students from the University of Sydney participated in the study as part of their course credit requirement. However, 10 participants’ data were not included as they did not finish the MSCEIT. A total of 53 participants (42 females, mean age = 20.58) were used in the current analyses. The selection criteria were as such: (1) be above 18 years old in age, (2) be moderately proficient in English, and (3) have no colour blindness. Power analysis was conducted using MorePower 6.0 with the specifications for a 2 (Memory Load) × 3 (Flanker Type) × 2 (SOA), with the desired power of 0.80 and an effect size of 0.14. The required sample size was 32. As with Study 2, the sample size requirements increased to 52 to account for the inclusion of the EI covariates. 

#### 4.1.2. Design, Materials, and Procedure

The design and protocol were largely identical to those employed in Studies 1 and 2. However, we varied the SOA of the flanking distractors in Study 3. Specifically, the flankers were either presented 300 ms or 1000 ms before the *n*-back probes. As before, these flankers continued to be present until the end of the trial. To keep the total number of trials for the entire procedure similar to Studies 1 and 2, we reduced the number of actual trials for each block to 24, with the same ratio of 1:2 for targets and non-targets. To compare between low and high memory load conditions with different SOAs for the distractors, we dropped the 2-back condition and had participants complete a 1-back and 3-back instead. Participants completed a total of 288 actual trials over 12 blocks. The experimental duration lasted for approximately 60 to 75 min.

### 4.2. Results

The scoring and analyses used were identical to Studies 1 and 2. The correlations between the branches of the MSCEIT in this study were not all correlated with each other (see Table 12). Branch 1: Perceiving Emotions was not significantly correlated with Branch 3: Understanding Emotions or Branch 4: Managing Emotions. Additionally, Branch 2: Using Emotions was not significantly correlated with Branch 3.

#### 4.2.1. Accuracy

The MANCOVA with Experiential EI and Strategic EI covariates revealed main effects of memory load, *F*(1, 50) = 84.768, partial-η^2^ = 0.629, Wilks’ Λ = 0.371, *p* < 0.001, and SOA, *F*(1, 50) = 6.924, partial-η^2^ = 0.122, Wilks’ Λ = 0.878, *p* = 0.011; but no main effects of flanker type, *F*(2, 49) = 1.099, partial-η^2^ = 0.043, Wilks’ Λ = 0.957, *p* = 0.341. The Flanker x SOA interaction was significant, *F*(2, 49) = 5.683, partial-η^2^ = 0.188, Wilks’ Λ = 0.812, *p* = 0.006 (see Table 13).

As expected, accuracy rates on a whole decreased significantly as memory load demands increased from 1-back to 3-back, regardless of SOA (*p* < 0.001*)*. The source of the 2-way interaction between SOA x Flanker was when the SOA duration was at 1000ms. At the longer SOA duration, the happy face flankers condition had significantly lower accuracy as compared to the shape flankers condition (*p* = 0.008). All other comparisons were not significant. Therefore, the interference effects of the emotional flankers were most evident when the distractor’s SOA was at the longest, although this was mainly due to the contributions of happy face distractors. In other words, a longer distractor SOA could elicit an emotional interference effect at the 3-back load condition but only for happy faces. This could partially explain why an emotional interference effect was not obtained at the 3-back level for Studies 1 and 2 in terms of accuracy rates. 

Regarding the covariates, there was a marginally significant interaction between SOA x Strategic EI, *F*(1, 50) = 3.851, partial-η^2^ = 0.072, Wilks’ Λ = 0.928, *p* = 0.055. The interaction term between Load x Strategic EI also reached near significance, *F*(1, 50) = 3.972, partial-η^2^ = 0.074, Wilks’ Λ = 0.926, *p* = 0.052. To explore this relationship, we correlated Strategic EI with overall accuracies for 1-back and 3-back conditions. Strategic EI was positively correlated with accuracies at the 1-back level, *r*(53) = 0.476, *p* < 0.001 and at the 3-back level, *r*(53) = 0.515, *p* < 0.001. In other words, a higher Strategic EI benefited accuracy performances at the 1-back and 3-back condition, which was a finding similar to Study 1. 

#### 4.2.2. Reaction Times for Hits

The MANCOVA yielded significant main effects of *n*-back load, *F*(1, 46) = 129.994, partial-η^2^ = 0.731, Wilks’ Λ = 0.269, *p* < 0.001, and of SOA, *F*(1, 46) = 22.398, partial-η^2^ = 0.327, Wilks’ Λ = 0.673, *p* < 0.001; but non-significant effects of flanker type, *F*(2, 45) = 0.053, partial-η^2^ = 0.017, Wilks’ Λ = 0.999, *p* = 0.819 (see Table 14). Regarding the MSCEIT EI covariates, there were no significant interaction effects between Strategic EI and all the other variables. The same was true for Experiential EI as a covariate as well. 

Importantly, there was a significant 3-way interaction effect of Memory Load x Flanker Type × SOA, *F*(2, 45) = 4.143, partial-η^2^ = 0.156, Wilks’ Λ = 0.844, *p* = 0.022. The source of the 3-way interaction was when the SOA differed within each Load × Flanker level. At the 1-back task, participants were significantly faster when the fearful face flankers were presented at 300 ms as compared to 1000 ms (*p* = 0.003). Additionally, participants performed significantly slower for shape flankers when the SOA was longer (*p* < 0.001). At the 3-back level, participants had significantly lower hit RTs when the fearful face flankers’ SOA was 1000 ms as compared to 300 ms (*p* = 0.017). The same pattern held for happy faces as well (*p* < 0.001). All other comparisons were not significant.

#### 4.2.3. Reaction Times for Correct Rejections

Similar to RTs for hits, the analyses yielded significant main effects of *n*-back load, *F*(1, 48) = 107.152, partial-η^2^ = 0.691, Wilks’ Λ = 0.309, *p* < 0.001, and of SOA, *F*(1, 48) = 38.511, partial-η^2^ = 0.445, Wilks’ Λ = 0.555, *p* < 0.001; but non-significant effects of flanker type, *F*(2, 47) = 0.0.98, partial-η^2^ = 0.004, Wilks’ Λ = 0.996, *p* = 0.907. Unlike RTs for hits, there was no significant interaction effect of Load x Flanker x SOA, *F*(2, 47) = 1.639, partial-η^2^ = 0.065, Wilks’ Λ = 0.935, *p* = 0.205 (see Table 15). 

Importantly, there was a significant interaction effect for Flanker Type x SOA x Experiential EI, *F*(2, 47) = 5.875, partial-η^2^ = 0.200, Wilks’ Λ = 0.800, *p* = 0.005. Correlational analyses indicated that Experiential EI was positively associated with RTs for correct rejections at the 3-back task with happy flankers (300 ms), *r*(53) = 0.291, *p* = 0.034. This suggests that participants with higher Experiential EI were much longer in rejecting the non-target probes when the flankers were presented 300 ms before the probes were centrally presented on the screen. 

### 4.3. Discussion for Study 3

At a longer SOA (1000 ms), an emotional interference effect was obtained at the 3-back level, although this was only for happy face distractors. Strategic EI was positively associated with overall accuracies at the 1-back and 3-back levels. This finding is similar to Study 1, in that a higher Strategic EI benefited accuracy performance for a cognitively challenging task. At least for correct rejection trials, Experiential EI was positively associated with response latencies at the 3-back level. While not fully replicating the findings of Study 2, the results in Study 3 also suggest that Experiential EI could be implicated when performance was measured using response latencies instead of accuracies. Such a notion would be complementary to Gutiérrez-Cobo’s et al. (2017a, 2017b) findings. 

## 5. General Discussion

The purpose of the present set of studies was to explore the relationships between affective and non-affective inhibition in the context of working memory updating. The second purpose was to explore how different aspects of ability EI contributed to these processes from an individual differences perspective. The final purpose was to explore whether a common but challenging cognition task, such as the *n*-back, could be used to study the contributions of ability EI on working memory updating and emotional inhibition. To answer these research questions, we employed the ability-based model of EI as measured by the MSCEIT (Mayer et al. 2003). In Study 1, we combined a standard letter *n*-back task with affective or non-affective flanking distractors. Affective flankers were either happy faces or fearful faces, while non-affective flankers were either shapes or letters. In Study 2, we dropped the letter flanker conditions as they appeared to be generating additional interference due to their similarity with the *n*-back probes. While this type of interference could be meaningful and influence performance (i.e., in a typical flanker task), it was not related to the non-emotional versus emotional aspects of the current *n*-back task. We also replaced the 0-back condition with a 1-back condition because participants were reaching max accuracy on the former. In Study 3, we varied the SOAs of flanking distractor by either 300 ms or 1000 ms. This was done to determine whether it was possible to elicit a stronger emotional effect at the 3-back level. 

### 5.1. Analysis 1: Affective Versus Non-Affective Flanker Distractors on N-back Performance

When considering *n*-back performance without any ability EI influences, the results indicate impaired accuracy rates for emotional face flankers. This was most evident in Study 1, participants had significantly lower accuracy rates at the 2-back level, and when presented with fearful faces than with neutral shape flankers. However, the emotional interference was not observed when memory load was high (3-back). This finding was surprising as it seemed counter-intuitive to the broader literature on working memory, specifically the impact of emotional valence on performance. For example, Grissmann et al. (2017) found that negative-affect stimuli had detrimental effects on accuracy rates and RTs. We had earlier hypothesized that the impact of emotional valence would be even more pronounced as memory load increased. However, the absence of an emotional interference effect could be incidentally due to increased load demands at the 3-back level. That is, increasing executive functioning demands might have obscured subtle interference effects generated from the emotional face flankers. Previous researchers had suggested that there could be a strong interplay of attentional demands and memory load on executive functioning processes (McCabe et al. 2010). For example, Kim et al. (2005) and de Liaño et al. (2010) found that interference effects from distractors could be attenuated under high working memory load, suggesting that under the right conditions affective flanker effects were nullified instead. In a more recent finding, Scharinger et al. (2015) found that increasing memory load resulted in increased activation on one’s attentional network, making task performance less susceptible to distractor information. 

In the context of Study 1, the increased activations of attention processes under high working memory load could have facilitated participants’ inhibitory control in the high memory (3-back) load conditions. This enabled participants to suppress the attention-grabbing nature of the emotional face flankers. The challenging demands of our EFNB task might have incidentally focused or narrowed participants’ controlled attention, thereby enhancing their attentional focus towards the central letter probes on the *n*-back task. Essentially, the increased working memory demands at the 3-back conditions acted as a shield against all distracting flankers, whether affective or not. In hindsight, Study 1 should have included a 1-back (low memory load) condition as a comparison group for emotional interference effects against the 2-back or 3-back condition. 

In study 2, we swapped the 0-back conditions with a 1-back variant. We replicated the emotional interference of fearful face flankers vis-à-vis neutral shape flankers at the 2-back level in terms of poorer accuracy rates. Again, we did not find a significant accuracy difference between emotional versus non-emotional distractors at the 3-back level. A much more interesting finding was that response latencies for hit trials (correct responses) were significantly faster for emotional faces (both happy and fearful) when compared to shape flankers at the 3-back level. Thus, while participants were perhaps equally accurate at the 3-back level regardless of flanker types, they were much quicker to respond when presented with emotional flankers. Emotional information promotes adaptive responses because it is vital for survival to quickly attend to or to avoid aversive stimuli (e.g., survival processing; Nairne et al. 2008). In the context of our present study, this automatic vigilance for emotional flankers could have incidentally sped up processing on the central task at the 3-back level. Perhaps participants actively avoided the emotion face flankers, which led to them focusing on the central letter probes instead. This attentional bias away from valenced stimuli also suggests an automatic mechanism that could modulate available cognitive resources away from aversive distractors in order to focus on the task at hand. 

Combining the results of Study 1 and 2, it appears that the task demands associated with increasing memory load could have ameliorated or facilitated the effects of emotional distractors (Scharinger et al. 2015). One reason for this might be because the distractors and memory load demands occurred at the same time in the *n*-back task. This in turn forced the cognitive system to prioritize one task over the other because attention and other executive control processes are limited in capacity (i.e., an argument similar to Load Theory; Lavie et al. 2014). To explore whether we could elicit an emotional interference effect at the 3-back level, the emotional face and neutral distractors were presented either 300 ms or 1000 ms before the *n*-back probes.

The results from Study 3 indicate that *n*-back accuracy rates were significantly impaired for happy face flankers (SOA: 1000 ms) when compared to neutral shape flankers. This emotional interference effect was obtained at the 3-back level. As such, it seems that briefly presenting the emotional distractors prior to the *n*-back probes could impair subsequent updating processes. This could explain why we did not obtain an emotional interference effect for accuracy rates at the 3-back level for Studies 1 and 2. When working memory demands are high, it may be necessary to pre-emptively present emotional flankers in order to generate affective interference for subsequent processing (Lavie et al. 2014).

### 5.2. Analysis 2: Individual Differences of EI Abilities

In contrast to Gutiérrez-Cobo et al. (2017b), we found that the Strategic EI facet on the MSCEIT contributed significantly to *n*-back performances regardless whether the WMC task was of a hot or cold variant (Study 1). The findings from Study 1 are also inconsistent with an earlier Gutiérrez-Cobo’s et al. (2017a) study, where they found that the Managing branch (i.e., a component under the Strategic EI facet) on the MSCEIT was correlated with the cognitive control of emotional information but not with non-emotional information. On the other hand, the results obtained Study 2 and Study 3 are complementary to those obtained in Gutiérrez-Cobo et al. (2017b). Specifically, these results suggested that Experiential EI would be more implicated when performance was measured using response latencies, whereas Strategic EI would be implicated when accuracy rates were utilized (as in Gutiérrez-Cobo’s et al. studies). 

Study 1 also provided evidence regarding the binary aspects of ability EI, specifically emotional perception (we refer to as Experiential EI) versus emotional reasoning (Strategic EI; Mayer et al. 2003). Emotional perception is reflective of the more basic level of emotional processing (e.g., perception of emotions in others), while emotional reasoning can be described as a set of online abilities of emotional reasoning (i.e., appropriately managing and using emotions to suit the contextual situation at hand). In other words, Experiential EI consists of abilities that use long-term emotional knowledge to accurately perceive and judge situations, while Strategic EI is the fluid and regulatory management of emotions towards fulfilling the central goal at the current moment. In Study 1, Strategic EI but not Experiential EI was the main contributing factor regarding participants’ accuracy rates on a challenging *n*-back task. 

One possible reason for Strategic EI modulating working memory performance at both neutral and emotional conditions may have to do with task difficulty. In Gutiérrez-Cobo and colleagues’ studies, they employed a traditional *n*-back task that did not use any flanking distractors and found that higher EI abilities significantly predicted participants’ performance on the “hot” (emotional face probes) *n*-back task but not on the “cool” (letter probes) *n*-back task. In our study, participants had to simultaneously inhibit the effects of the flankers and to perform an updating task. As the memory load increased, it placed additional burden on the cognitive system for maintaining and updating the target probes. This could have substantially increased the EFNB task’s difficulty as compared to the standard working memory tasks employed in Gutiérrez-Cobo’s et al. (2017b) study. Additionally, they only used a 2-back task, whereas in our current studies we had participants complete a 3-back variant as well. The 3-back task is much more cognitively challenging and requires a considerable amount of mental effort (Jaeggi et al. 2010). In our current study, perhaps the 2-back and 3-back load conditions had elevated processing demands associated with concurrently inhibiting the distractor flankers while having to update sequential letter probes. If so, Strategic EI abilities (as measured using the MSCEIT) should be more utilized in such cognitively demanding situations. 

Studies 2 and 3 were conducted to determine if we could replicate the effects of Strategic EI on the emotional *n*-back task. At the same time, we varied different parameters of the task in order to elicit stronger emotional effects. This would answer several questions, including whether such a cognition task could be used to examine individual differences of ability EI. Additionally, we attempted—in Study 3—to replicate the pattern of findings obtained in Studies 1 and 2. We were also intrigued by the significant interaction between memory load and Experiential EI in Study 1, though none of the correlation pairs were significant. The pattern of results suggests that Strategic EI contributed to accuracy performance while Experiential EI was more about the speed of which the task could be completed.

Contrary to our expectations, we could not replicate the contributions of Strategic EI on accuracy rate for the emotional *n*-back task used in Study 2. One possible explanation was that the contributions of EI to cognition tasks such as the *n*-back were not wholly stable. Importantly, the results from Study 2 indicated that Experiential EI was significantly and positively associated with RTs for hits and correct rejections for both fearful and happy face flankers. In other words, high scorers for Experiential EI could have incidentally performed the *n*-back task much slower as compared to low scorers of Experiential EI, or vice versa. This pattern of results was seen in Study 3 as well. Austin (2009) found that participants higher in ability EI (as measured using the Situational Judgement Test) were significantly slower to respond to difficult test items. In the context of Studies 2 and 3, it seemed that participants with higher Experiential EI succeeded in performing on the *n*-back task by spending more time before responding accurately. 

Taken together, the current set of studies suggest that the contributions of ability EI could possibly differ on whether Strategic EI or Experiential EI are used. Ability EI is not a univariate construct, with multiple abilities supporting or underlying this construct. In terms of managing one’s emotional or motivational states to get the task done as accurately as possible, Strategic EI appears to play a more important role. Managing one’s emotions is a key component of Strategic EI and several studies have demonstrated that this ability is useful for improving task performance (Checa and Fernández-Berrocal 2015). On the other hand, abilities underlying Experiential EI could be contributing to the speed in performing a challenging cognitive task (Studies 1, 2, and 3). If true, these dual aspects of the MSCEIT may provide interesting avenues of research into their impact on cognition.

### 5.3. Limitations and Future Research

A limitation of the current study is that we focused only on the performance-based ability model as measured by the MSCEIT but did not include general (or cognitive) intelligence. Previous studies examining EI abilities and executive functioning also did not employ measures of general intelligence (Gutiérrez-Cobo et al. 2017b). The ability model is the best researched model of EI, with a relatively clear demarcation of the related abilities. Importantly, the ability model emphasises that EI is a broad class of intelligence and one that focuses predominantly on emotional information processing (Mayer et al. 2016). In our study, Strategic EI was implicated when working on an updating task and with the need to inhibit or resolve the interference generated from the flanking distractors. However, several abilities underlying cognitive intelligence (e.g., fluid reasoning, working memory capacity) also play a substantial role in distractor-conflict resolution and updating (Kane et al. 2007). As such, it will be worthwhile for future researchers to incorporate a measure of general intelligence or other cognitive variables when examining the contributions of EI abilities towards participants’ performance on an emotional *n*-back task. 

A second limitation is the smaller sample size used in our set of studies when compared to previous ability EI studies and cognition. For example, Gutiérrez-Cobo et al. (2017b) had 203 participants complete the MSCEIT, two other EI measures, and a 2-back task. In their earlier study (Gutiérrez-Cobo et al. 2017a), 199 undergraduate participants completed the same number of EI measures as well as a hot and cold go/no-go task. In comparison, we utilized a quarter of this number of participants in each of our studies reported here. By design and from the power analyses, our studies were sufficiently powered. Increasing the sample size might lead to the point at which many relationships between the MSCEIT and *n*-back performances might be statistically significant, because “everything correlates to some extent with something else” (Meehl 1990, p. 204). We sought to use a simpler design and one that allows for quick replicability by future researchers in the intersection between EI and cognition.

A third limitation is perhaps by design, in that we a priori decided on using the two binary facets of the MSCEIT rather than the individual branches. As noted earlier in the literature review, a number of studies had provided contrary evidence for the four-factor model of the MSCEIT (Fiori and Antonakis 2011). Mayer et al. (2003)—the authors of the original MSCEIT—recommended the use of Total EI (i.e., combination of all four branches) as the test measures “one unique source of variation” (p. 508). Other researchers (e.g., Fan et al. 2010) suggested that a three-factor model fits better, as they found that Branch 1 and Branch 2 were highly correlated (*r* = 0.90). We acknowledge that the question of whether to use the individual branches or the composite facets is a research question by itself. Nevertheless, we decided to take a middle ground approach by primarily focusing on the facets. We also opted to use the facets as a means of streamlining the analyses based on the experimental design employed in the current studies. 

Finally, as noted earlier, the effects of ability EI might not be stable when measured using a cognitive task such as the *n*-back. To date, only Gutiérrez-Cobo et al. (2017b) had examined the contributions of ability EI using an updating task and no further replication attempts were made by them or other researchers. In the current set of studies, we had attempted to explore these contributions using the *n*-back task but also with variations to the protocol. Future studies can vary different parameters of the *n*-back task to better demonstrate whether ability EI provides performance affordances (for accuracy rates) or answer why it substantially increases response latencies. 

### 5.4. Conclusions

In conclusion, our current study highlighted the contributions of ability EI to working memory updating. Different EI abilities can be important individual differences that influence updating performances as task complexity changes. We also noted the similarities and differences between inhibition of affective and non-affective distractors when presented in the *n*-back task. Finally, our study established an experimental approach to examine EI abilities with cognition, especially in terms of accuracy performance and response latencies. This will open up new and exciting possibilities in understanding the cognitive underpinnings of EI, especially in relation to the other frequently postulated (but distinct) executive functions of inhibition, switching, and updating. Importantly, the current study contributed to answering the question of whether EI can truly be classified as a set of abilities or emotional competencies that can be utilized when the situation requires it. Our studies suggest that both Strategic EI and Experiential EI have important roles to play when working on a cognitively demanding task. Understanding the cognitive processes underlying EI abilities will be crucial in determining whether such abilities can be improved upon.

## Figures and Tables

**Table 1:** Correlations between Mayer-Salovey-Caruso Emotional Intelligence Test (MSCEIT) scores.

	1	2	3	4	5	6	7
1. Perceiving (B1)	1						
2. Using (B2)	0.450 **	1					
3. Understanding (B3)	0.393 **	0.388 **	1				
4. Managing (B4)	0.222 *	0.391 **	0.352 **	1			
5. Experiential EI	0.915 **	0.762 **	0.460 **	0.339 **	1		
6. Strategic EI	0.398 **	0.484 **	0.867 **	0.744 **	0.507 **	1	
7. Total EI	0.745 **	0.710 **	0.769 **	0.634 **	0.857 **	0.874 **	1

Note. B = Branch; EI = emotional intelligence. ** Correlation is significant at the 0.01 level (2-tailed), * Correlation is significant at the 0.05 level (2-tailed).

**Table 2:** Means and SDs of participants’ accuracy rates on the Emotional Flanker N-back (EFNB) task (Study 1).

Flanker Type	Memory Load					
	0-Back		2-Back		3-Back	
	*M*	*SD*	*M*	*SD*	*M*	*SD*
Fearful	0.966	0.062	0.829	0.140	0.786	0.157
Happy	0.969	0.060	0.846	0.151	0.783	0.157
Letters	0.960	0.074	0.848	0.145	0.792	0.154
Shapes	0.959	0.086	0.873	0.141	0.798	0.141
**Total**	0.963	0.070	0.849	0.144	0.790	0.152

**Table 3:** N-back accuracy rates at each load level for different Strategic EI groups.

Memory Load	Low Strategic EI ^a^		Mid Strategic EI ^b^		High Strategic EI ^c^	
	*M*	*SD*	*M*	*SD*	*M*	*SD*
0-back	0.939	0.078	0.967	0.042	0.985	0.027
2-back	0.784	0.158	0.864	0.096	0.899	0.086
3-back	0.743	0.134	0.782	0.123	0.845	0.105

a = 28 participants; b = 29 participants; c = 28 participants.

**Table 4:** Descriptive statistics of participants’ mean reaction times for Hits (before log-transformed).

Flanker Type	Memory Load					
	0-Back		2-Back		3-Back	
	*M*	*SD*	*M*	*SD*	*M*	*SD*
Fearful	631.013	167.754	804.412	288.025	919.637	309.028
Happy	636.265	192.034	871.634	322.036	925.887	326.140
Letters	657.526	200.719	890.274	310.280	950.037	349.628
Shapes	623.111	172.265	832.901	297.307	926.040	311.174
**Total**	636.979	183.193	849.805	304.412	930.400	323.993

**Table 5:** Estimates of hit reaction times (before log-transformed) for each flanker type.

Flanker Type	*M*	*SD*
Fearful	785.02	254.94
Happy	811.26	280.07
Letter	832.61	286.88
Shape	794.02	260.25

**Table 6:** Descriptive statistics of participants’ mean reaction times for correct rejections (before log-transformed) for Study 1.

Flanker Type	Memory Load					
	0-Back		2-Back		3-Back	
	*M*	*SD*	*M*	*SD*	*M*	*SD*
Fearful	657.74	188.09	851.54	271.25	918.86	315.17
Happy	661.76	207.96	877.86	293.26	932.92	332.73
Letters	681.68	173.45	903.01	308.48	939.73	331.09
Shapes	643.23	163.95	866.84	296.44	910.45	317.24
**Total**	661.10	183.36	874.81	292.36	925.49	324.06

**Table 7:** Estimates of Reaction Times for correct rejection (before log-transformed) for each flanker type.

Flanker Type	*M*	*SD*
Fearful	809.01	258.17
Happy	824.18	277.98
Letter	841.47	271.01
Shape	806.84	259.21

**Table 8:** Correlations between MSCEIT scores for Study 2.

	1	2	3	4	5	6	7
1. Perceiving (B1)	1						
2. Using (B2)	0.467 **	1					
3. Understanding (B3)	0.126	0.187	1				
4. Managing (B4)	0.401 **	0.220	0.355 *	1			
5. Experiential EI	0.941 **	0.715 **	0.198	0.406 **	1		
6. Strategic EI	0.262 *	0.204	0.894 **	0.724 **	0.308 *	1	
7. Total EI	0.754 **	0.593 **	0.646 **	0.690 **	0.823 **	0.781 **	1

**. Correlation is significant at the 0.01 level (2-tailed); *. Correlation is significant at the 0.05 level (2-tailed).

**Table 9:** Means and SDs of participants’ accuracy rates for all conditions for Study 2.

Flanker Type	Memory Load					
	1-Back		2-Back		3-Back	
	*M*	*SD*	*M*	*SD*	*M*	*SD*
Fearful	0.937	0.061	0.852	0.103	0.778	0.115
Happy	0.919	0.132	0.868	0.094	0.789	0.114
Shapes	0.934	0.084	0.888	0.092	0.778	0.078
**Total**	0.930	0.092	0.869	0.096	0.782	0.102

**Table 10:** Descriptive statistics of participants’ mean reaction times for hits (before log-transformed) for Study 2.

Flanker Type	Memory Load					
	1-Back		2-Back		3-Back	
	*M*	*SD*	*M*	*SD*	*M*	*SD*
Fearful	618.83	164.33	752.66	247.05	752.77	252.43
Happy	634.55	203.31	716.89	236.57	760.82	287.62
Shapes	589.48	156.78	747.76	252.52	834.71	273.31
**Total**	614.29	174.81	739.10	245.38	782.77	271.12

**Table 11:** Descriptive statistics of participants’ mean reaction times for correct rejections (before log-transformed) for all conditions in Study 2.

Flanker Type	Memory Load					
	1-Back		2-Back		3-Back	
	*M*	*SD*	*M*	*SD*	*M*	*SD*
Fearful	617.30	176.33	762.83	282.77	753.98	293.66
Happy	628.19	201.86	791.33	319.41	760.90	302.13
Shapes	596.29	169.48	773.05	295.68	769.97	268.70
**Total**	613.93	182.56	775.74	299.29	761.62	288.16

**Table 12:** Correlations between MSCEIT scores (Study 3).

	1	2	3	4	5	6	7
1. Perceiving (B1)	1						
2. Using (B2)	0.456 **	1					
3. Understanding (B3)	−0.002	0.159	1				
4. Managing (B4)	0.234	0.438 **	0.276 *	1			
5. Experiential EI	0.917 **	0.755 **	0.073	0.374 **	1		
6. Strategic EI	0.118	0.301 *	0.888 **	0.663 **	0.228	1	
7. Total EI	0.627 **	0.636 **	0.638 **	0.682 **	0.735 **	0.812 **	1

**. Correlation is significant at the 0.01 level (2-tailed); *. Correlation is significant at the 0.05 level (2-tailed).

**Table 13:** Means and SDs of participants’ accuracy rates for Study 3.

	Memory Load							
	1-Back				3-Back			
Stimulus Onset Asynchrony	300 ms		1000 ms		300 ms		1000 ms	
Flanker	*M*	*SD*	*M*	*SD*	*M*	*SD*	*M*	*SD*
Fearful Faces	0.919	0.130	0.943	0.102	0.829	0.129	0.831	0.121
Happy Faces	0.934	0.079	0.937	0.101	0.813	0.148	0.815	0.131
Shapes	0.925	0.112	0.965	0.044	0.796	0.142	0.866	0.132
**Total**	0.926	0.107	0.948	0.082	0.813	0.140	0.837	0.128

**Table 14:** Means and SDs of participants’ reaction times for hit trials for Study 3.

Stimulus Onset Asynchrony	Memory Load							
	1-Back				3-Back			
	300 ms		1000 ms		300 ms		1000 ms	
Flanker	*M*	*SD*	*M*	*SD*	*M*	*SD*	*M*	*SD*
Fearful Faces	633.56	171.58	688.95	164.81	881.96	308.16	972.77	276.93
Happy Faces	655.07	187.27	680.25	145.72	845.23	277.39	1012.97	318.05
Shapes	613.81	136.87	709.20	199.92	895.94	287.49	939.05	289.56
**Total**	634.15	165.24	692.80	170.15	874.38	291.02	974.93	294.84

**Table 15:** Means and SDs of participants’ correct rejection reaction times for Study 3.

Stimulus Onset Asynchrony	Memory load							
	1-Back				3-Back			
	300 ms		1000 ms		300 ms		1000 ms	
Flanker	*M*	*SD*	*M*	*SD*	*M*	*SD*	*M*	*SD*
Fearful Faces	621.15	160.60	753.67	195.40	829.59	266.15	925.71	279.82
Happy Faces	628.89	146.48	721.45	143.13	810.75	280.17	945.79	249.75
Shapes	629.62	151.70	722.16	189.01	850.04	301.12	902.88	273.04
**Total**	626.55	152.93	732.43	175.85	830.13	282.48	924.79	267.53

## Footnotes

1 We followed the suggestions by DeCoster et al. (2011) to first analyse our data continuously (e.g., using ANCOVA) and then to use the means obtained from artificially categorizing EI scores to interpret the interactions between Strategic EI and Memory Load.

2 Unlike the typical university sample pool, Prolific is a web-based research platform that allows for more gender-balanced sampling. As such, the gender breakdown in Study 2 was approximately 1:1, whereas Study 1 used a psychology undergraduate sample pool where the female-to-male ratio is often skewed towards the former. Nevertheless, the ANOVAs with gender as a fixed factor and the dependent variables as Experiential EI, Strategic EI, and Total EI revealed no significant differences in Study 2.

3 We also correlated Branch 4: Managing Emotions with RTs for Correct Rejection trials. None of the associations were significant.
